# A Survey of Clinical Practices for Hepatocellular Carcinoma Among Experts at Tertiary Hospitals in China From 2020 to 2021

**DOI:** 10.1002/cai2.70006

**Published:** 2025-04-07

**Authors:** Hong Zhao, Yilei Mao, Hongguang Wang, Aiping Zhou, Zhengqiang Yang, Yue Han, Gong Li, Xinyu Bi, Chunyi Hao, Xiaodong Wang, Jun Zhou, Chaoliu Dai, Feng Wen, Jingdong Zhang, Ruibao Liu, Tao Li, Lei Zhao, Zuoxing Niu, Tianfu Wen, Qiu Li, Hongmei Zhang, Xiaoming Chen, Minshan Chen, Ming Zhao, Yajin Chen, Jun Yu, Jie Shen, Xiangchen Li, Lianxin Liu, Zhiyong Huang, Wei Zhang, Feng Shen, Weiping Zhou, Zhengang Yuan, Jian Zhai, Ningling Ge, Yongjun Chen, Huichuan Sun, Jianqiang Cai

**Affiliations:** ^1^ Department of Hepatobiliary Surgery, National Cancer Center/National Clinical Research Center for Cancer/Cancer Hospital Chinese Academy of Medical Sciences and Peking Union Medical College Beijing China; ^2^ Department of Liver Surgery, Peking Union Medical College (PUMC) Hospital PUMC and Chinese Academy of Medical Sciences (CAMS) Beijing China; ^3^ Department of Medical Oncology, National Cancer Center/National Clinical Research Center for Cancer/Cancer Hospital Chinese Academy of Medical Sciences and Peking Union Medical College Beijing China; ^4^ Department of Interventional Radiology, National Cancer Center/National Clinical Research Center for Cancer/Cancer Hospital Chinese Academy of Medical Sciences and Peking Union Medical College Beijing China; ^5^ Department of Radiation Oncology, Beijing Tsinghua Changgung Hospital (BTCH) School of Clinical Medicine, Tsinghua University Beijing China; ^6^ Key Laboratory of Carcinogenesis and Translational Research (Ministry of Education), Sarcoma Center Peking University Cancer Hospital & Institute Beijing China; ^7^ Departments of Interventional Oncology Peking University Cancer Hospital & Institute Beijing China; ^8^ Department of Medical Oncology Peking University Cancer Hospital & Institute Beijing China; ^9^ Department of General Surgery Shengjing Hospital of China Medical University Shenyang Liaoning China; ^10^ Department of Radiology Shengjing Hospital of China Medical University Shenyang Liaoning China; ^11^ Medical Oncology Department of Gastrointestinal Cancer Liaoning Cancer Hospital & Institute, Cancer Hospital of China Medical University Shenyang Liaoning China; ^12^ Interventional Radiological Department Harbin Medical University Cancer Hospital Harbin Heilongjiang China; ^13^ Department of General Surgery, Qilu Hospital The Second Hospital of Shandong University Jinan Shandong China; ^14^ Department of Hepatobiliary Surgery Shandong Cancer Hospital Affiliated to Shandong First Medical University and Shandong Academy of Medical Science Jinan Shandong China; ^15^ Department of Gastroenterology, Ward 2, Shandong Cancer Hospital and Institute Shandong First Medical University Jinan Shandong China; ^16^ Department of Liver Surgery, West China Hospital Sichuan University Chengdu Sichuan China; ^17^ Cancer Center, West China Hospital Sichuan University Chengdu Sichuan China; ^18^ Department of Clinical Oncology, Xijing Hospital The Air Force Military Medical University Xi'an Shaanxi China; ^19^ Department of Interventional Radiology Guangdong Provincial People's Hospital Guangzhou Guangdong China; ^20^ Department of Liver Surgery Sun Yat‐sen University Cancer Center, State Key Laboratory of Oncology in South China, Collaborative Innovation Center for Cancer Medicine Guangzhou Guangdong China; ^21^ Department of Minimally Invasive Interventional Therapy, Liver Cancer Study and Service Group Sun Yat‐sen University Cancer Center Guangzhou Guangdong China; ^22^ Department of Hepatobiliary Surgery, Sun Yat‐sen Memorial Hospital Sun Yat‐sen University Guangzhou Guangdong China; ^23^ Department of Hepatobiliary and Pancreatic Surgery, The First Affiliated Hospital Zhejiang University School of Medicine Hangzhou Zhejiang China; ^24^ Department of Oncology The Affiliated Drum Tower Hospital of Nanjing University Medical School Nanjing Jiangsu China; ^25^ Hepatobiliary Center The First Affiliated Hospital of Nanjing Medical University Nanjing Jiangsu China; ^26^ Department of Hepatobiliary Surgery, The First Affiliated Hospital of USTC, Division of Life Sciences and Medicine University of Science and Technology of China Hefei Anhui China; ^27^ Hepatic Surgery Center, Tongji Hospital, Tongji Medical College Huazhong University of Science and Technology Wuhan Hubei China; ^28^ Department of Hepatobiliary Surgery, Eastern Hepatobiliary Surgery Hospital Second Military Medical University (Naval Medical University) Shanghai China; ^29^ Department of Oncology, Eastern Hepatobiliary Surgery Hospital Second Military Medical University Shanghai China; ^30^ Department II of Interventional Radiology Eastern Hepatobiliary Surgery Hospital Shanghai China; ^31^ Department of Hepatic Oncology, Zhongshan Hospital, Liver Cancer Institute and Key Laboratory of Carcinogenesis and Cancer Invasion Fudan University Shanghai China; ^32^ Department of General Surgery, Ruijin Hospital Shanghai Jiao Tong University School of Medicine Shanghai China; ^33^ Department of Liver Surgery and Transplantation, Liver Cancer Institute and Zhongshan Hospital Fudan University Shanghai China

**Keywords:** adjuvant therapy, downstaging/conversion therapy, immunotherapy, neoadjuvant therapy, TKI

## Abstract

**Background:**

Hepatocellular carcinoma (HCC) is the second leading cause of cancer‐related death in China. The rapid progress in systemic therapies has led to the approval of many therapeutic methods that have quickly changed clinical guidelines and practices. Because of the high heterogeneity of HCC, there are still some gaps between the guidelines and real‐world clinical practice. The present study surveyed experts in China to investigate the current treatment concepts and clinical practice regarding HCC.

**Methods:**

A questionnaire survey on the treatment concepts and clinical practice of HCC was administered to 310 experts with senior professional titles in 2020 and 312 experts in 2021. The results were analyzed and compared.

**Results:**

For treating patients with resectable HCC, 28% of hepatobiliary surgeons indicated neoadjuvant therapy, and 7% chose systemic therapy ± locoregional therapy as 1 L therapy in 2021 compared with 20% and 1% in 2020. More experts chose adjuvant treatment within 1 month in 2021 compared with 2020, and 6 months and 12 months were the leading choices for the duration of adjuvant treatment. In 2021, 79% of surgeons and 19% of interventionalists were willing to conduct downstaging/conversion therapy for patients with potentially resectable HCC, and 78% chose tyrosine kinase inhibitors (TKI) + immunotherapy (IO) + locoregional therapy for cases in which R0 resection could not be achieved. For completely unresectable HCC, more experts preferred TKI + IO‐based therapy as 1 L therapy in 2021 compared with 2020 (78% vs. 55%). The proportion of experts who indicated TKI + IO‐based therapy as 2 L therapy increased from 32% in 2020 to 40% in 2021.

**Conclusion:**

The survey results indicated that in 2021, compared with 2020, more experts opted to administer IO + TKI for the treatment of liver cancer, and more experts and patients were willing to participate in clinical research.

## Graphical abstract

This study surveyed Chinese experts to investigate current treatment approaches for HCC. The nationwide questionnaires in 2020 and 2021 revealed increasing use of neoadjuvant, adjuvant, and combination systemic therapies like tyrosine kinase inhibitors plus immunotherapy for different HCC stages. The results highlight evolving real‐world clinical practice patterns rapidly adapting to new therapeutic options and guidelines for this highly heterogeneous cancer.

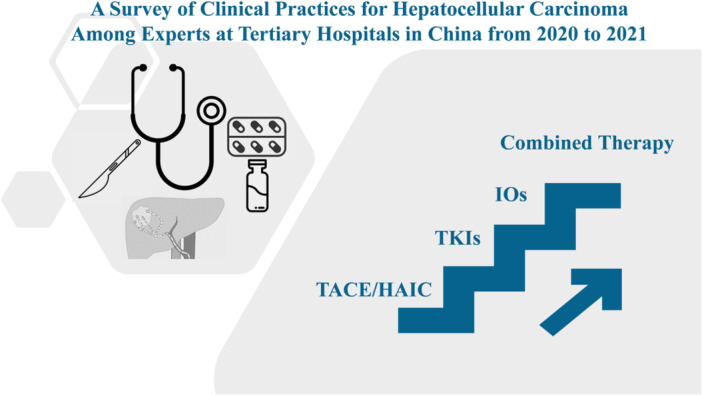

**Abbreviations**
HCC: hepatocellular carcinoma
IO: immunotherapy
ORR: objective response rate
OS: overall survival
TACE: transcatheter arterial chemoembolization
TKI: tyrosine kinase inhibitor
1L: first‐line
2L: second‐line

## 1 Introduction

Hepatocellular carcinoma (HCC) is the main type of primary liver cancer and one of the most common malignant tumors in China [1]. Surgical resection is the main treatment for HCC and can lead to long‐term survival of patients [2]. Because of the difficulty in diagnosis and rapid progression of HCC, many patients are diagnosed at the middle or advanced stage, and these patients are not eligible for radical surgical resection [3].

As a result of rapid progress over the past few years, combined targeting and immunotherapy‐based therapies have provided new treatment options for advanced HCC [4]. In the 2020 Chinese Society of Clinical Oncology Immunotherapy immune checkpoint inhibitor clinical practice, targeted immunotherapy was indicated as the preferred systemic treatment for patients with advanced unresectable HCC [5]. In recent years, HCC treatment guidelines from various countries have recommended targeted immunotherapy as the preferred treatment option for advanced unresectable HCC [6, 7, 8]. The development of systemic therapy has influenced the clinical practice guidelines and consensus of perioperative treatment. Preoperative treatment of HCC includes neoadjuvant therapy for resectable liver cancer and downstaging/conversion therapy for unresectable HCC [4]. Neoadjuvant therapy is suitable for patients with liver cancer that is technically resectable and with high‐risk factors for recurrence [9, 10]. In recent years, comprehensive adjuvant therapy, such as immunotherapy and molecular multi‐target antineoplastic drugs, has achieved remarkable curative effects and improved the survival and quality of life of HCC patients. Through systematic treatment, unresectable HCC has been converted to resectable HCC [11, 12, 13]. Moreover, personalized management strategies including converse therapeutic hierarchy, in which therapies are ordered in accordance with conversion abilities or adjuvant abilities (i.e., from systemic therapy to surgery), have been established [14]. With the results of many clinical studies with high levels of evidence, the guidelines and expert consensus are being updated. Therefore, conducting a survey on the current treatment concepts and clinical practice of HCC in China is urgent.

In this study, we administered a clinical questionnaire survey on the treatment of HCC to experts in tertiary hospitals in China in 2020 and 2021. The survey evaluated the treatment concepts and clinical practice for HCC.

## 2 Methods

### 2.1 Selection of Hospitals and Respondents

The distribution of medical resources throughout China is relatively uneven. The proportion of doctors with a high academic background in tertiary hospitals is high; these doctors have substantial experience in HCC diagnosis and treatment. Also, doctors in tertiary hospitals have more opportunities to carry out Phase III clinical research and can learn and practice the progress in the treatment field at home and abroad at the first time. Experts in tertiary hospitals are also responsible for transmitting new treatment concepts and technologies to doctors in lower‐level hospitals, playing the role of trendsetters in the industry. Therefore, doctors at the expert level in tertiary hospitals who were senior associate professors or professors were selected for this survey.

To obtain information on the changes and trends of experts' ideas promptly, we conducted surveys in the form of questionnaires from 2020 to 2021. We searched PubMed and other databases and found no questionnaire with relevant content, so we designed the questionnaire on the basis of the progress of the field and hot issues.

All experts were registered members of the Liver Cancer Branch of the Chinese Association for the Promotion of Medicine and had senior professional titles. WeChat was used to conduct the survey and collect the questionnaire results. All experts were provided informed consent, volunteered to participate, and agreed to the use of the collected data for scientific purposes. The questionnaire was a survey administered to experts and did not address patient data; individual patient data were not used in this study.

### 2.2 Survey Content

We designed the questionnaire on the basis of the newest clinical practice guidelines and consensus. The questionnaire addressed the following areas: (1) basic information of the therapeutic center (city, hospital, department, and proportions of patients with resectable, potentially resectable, and unresectable HCC); (2) treatment concepts and clinical practice of resectable HCC patients (proportion of patients that underwent neoadjuvant and adjuvant therapy, preferred regimen and reason, timing of adjuvant therapy after operation, and duration); (3) treatment concept and clinical practice of unresectable HCC patients (proportion of potential downstaging/conversion HCC patients, preferred treatment options and reasons, proportion of cases of successful conversions and criteria, interval between successful conversion and surgery, proportion of completely unresectable HCC patients, preferred first‐line (1 L) treatment choice and reasons, preferred second‐line (2 L) treatment options and reasons, the subsequent treatment options after progress on tyrosine kinase inhibitors (TKI) combined with immunotherapy (IO) and the proportion of cases that received TKI + IO as 2 L treatment); and (4) changes in the treatment concepts and clinical practice for HCC between 2020 and 2021.

### 2.3 Quality Control of the Questionnaire Results

Most dimensions of the questionnaire were designed as single‐choice questions, with only one best answer selected to ensure consistency. Multiple choices were set for fewer questions.

### 2.4 Statistical Analysis

Descriptive statistics are presented as frequencies and percentage distributions for the categorical data. Survey results were analyzed using Microsoft Excel.

## 3 Results

### 3.1 Basic Information and the Distribution of Respondents

We administered the survey questionnaire to 385 experts from 103 tertiary hospitals, which included 19 specialized hospitals and 84 comprehensive hospitals in China's provincial capitals or municipalities directly under the central government. Of the 385 surveyed experts, 312 and 310 responded in 2020 and 2021, with a response rate of 80% (310/385) and 81% (312/385), respectively. All respondents were experts in liver cancer treatment and had senior professional titles, including associate professor and professor, accounting for 18% (56/310) and 82% (254/310) of the overall group in 2020, respectively, and 16% (50/312) and 84% (262/312) in 2021, respectively. The educational background, professional title, department, hospital level, and clinical experience of the experts who participated in the survey in 2020 and 2021 were similar. In 2020, 97% (209/310) of the participating experts were from provincial capitals and municipalities directly under the central government. In 2021, this proportion was 92% (287/312). In 2020, 22% (67/310) of the experts were from specialized hospitals, while the rest were from comprehensive hospitals. In 2021, 20% (62/312) of the experts were from specialized hospitals. Most of the experts were from the Department of Hepatobiliary Surgery, accounting for 52% in 2020 and 50% in 2021. The next largest group was from the Department of Medical Oncology, accounting for 25% in 2020 and 29% in 2021. The remaining experts were from departments such as Interventional Radiology or Radiation Oncology.

### 3.2 Current Treatment Concepts and Clinical Practice for Resectable HCC

**Figure 1:**
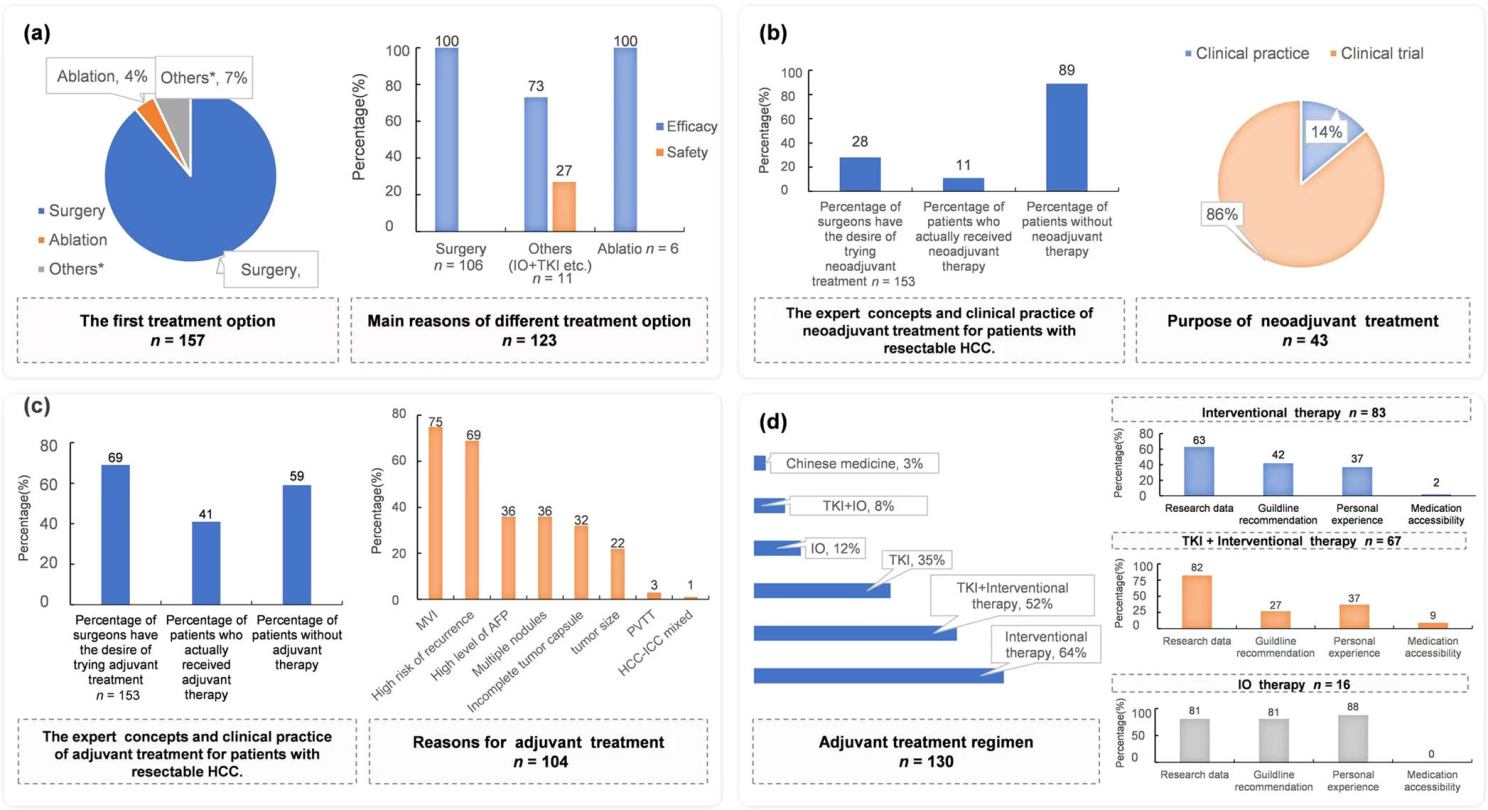
The treatment concepts and clinical practice for resectable HCC in 2021. (a) The first choice and cause of treatment for patients with resectable HCC. Others*: IO + TKI/IO + TKI + Interventional therapy/TKI + Interventional therapy/IO + Interventional therapy. (b) The expert concepts and clinical practice of neoadjuvant treatment for patients with resectable HCC. (c) Adjuvant therapy and patient profile of resectable HCC. (d) Adjuvant treatment regimen and consideration for resectable HCC. AFP, alpha‐fetoprotein; HCC, hepatocellular carcinoma; IO, immunotherapy; MVI, microvascular invasion; PVTT, portal vein tumor thrombosis; TKI, tyrosine kinase inhibitors.

The survey results showed that 89% of the hepatobiliary surgeons (140/157) considered surgical resection as the primary choice for resectable HCC patients, and the curative effect was the main reason for experts to select surgery (Figure 1a). Approximately 28% of hepatobiliary surgeons would select neoadjuvant therapy for resectable HCC patients, and 7% would select systemic therapy ± locoregional therapy. In practical application (in 2011), the neoadjuvant therapy was ultimately administered by 11% of experts (*n* = 43).

Neoadjuvant therapy was mainly used for clinical research rather than clinical practice (Figure 1b). Approximately 69% of hepatobiliary surgeons may use postoperative adjuvant therapy for resectable HCC patients. In clinical practice, approximately 41% (*n* = 106) of patients received adjuvant therapy after the operation. Patients with microvascular invasion were the primary targets for adjuvant therapy (Figure 1c). TKI + interventional therapy as the treatment option increased from 31% in 2020 to 52% in 2021. As shown in Figure 1d, 64% of experts considered interventional therapy as an adjuvant treatment regimen, followed by TKI + interventional therapy (52%), TKI therapy (35%), and IO therapy (12%). The experts indicated that research data were the primary consideration for their choice of interventional therapy and TKI + interventional therapy; personal experience was the primary consideration for their choice of IO therapy.

### 3.3 Current Treatment Concepts and Clinical Practice for Unresectable HCC

**Figure 2:**
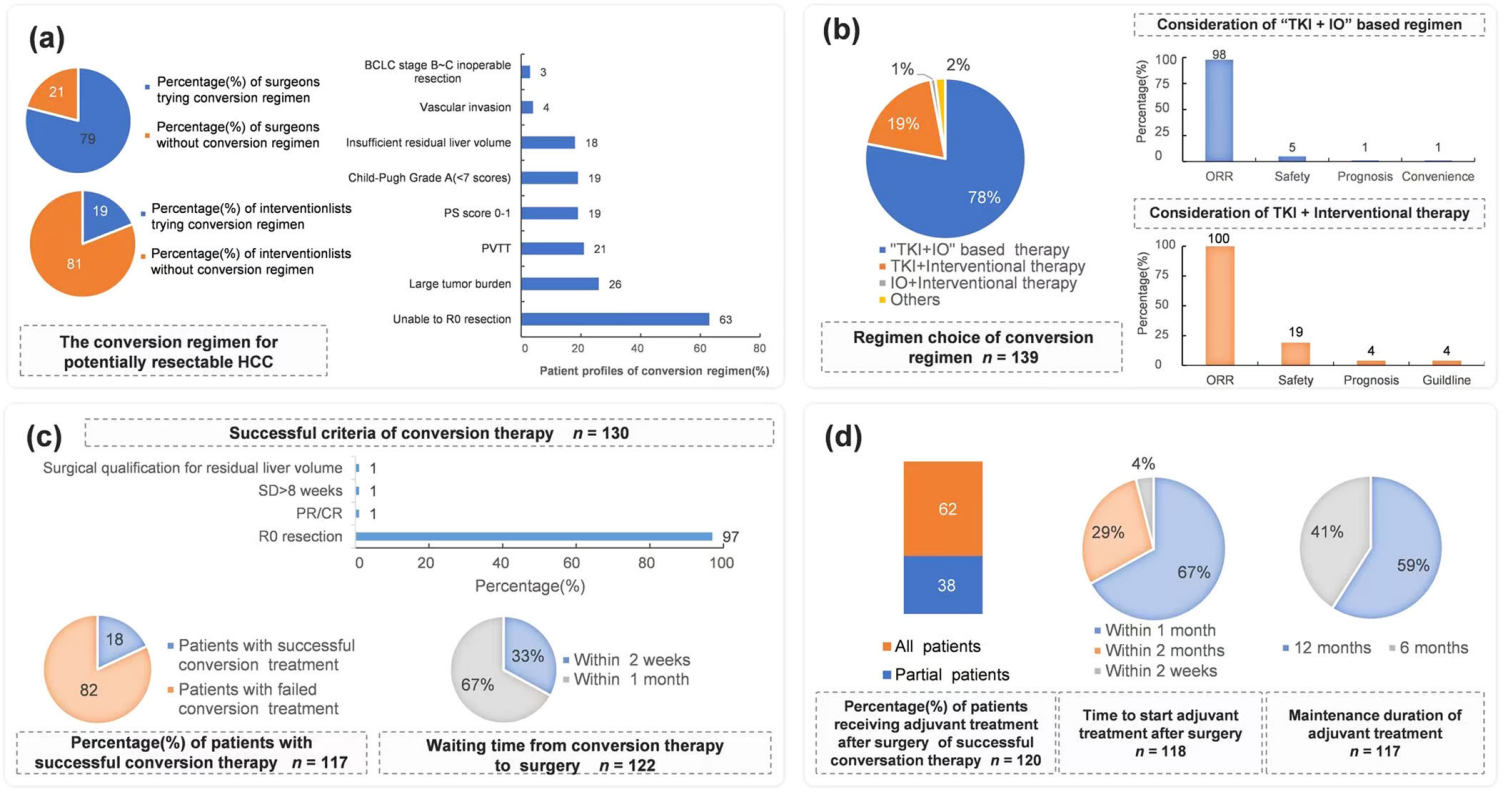
The treatment concepts and clinical practice for unresectable HCC. (a) The Down‐staging/conversion regimen for potentially resectable HCC and patient profiles of down‐staging/conversion regimen. (b) The down‐staging/conversion regimen choice and consideration of the experts. (c) Success criteria and successfully down‐staged portion of patients for potentially resectable HCC. (d) The treatment concepts and clinical practice of adjuvant treatment for potentially resectable HCC. CR, complete response; HCC, hepatocellular carcinoma; IO, immunotherapy; ORR, objective response rate; PR, partial response; PVTT, portal vein tumor thrombosis; SD, stable disease; TKI, tyrosine kinase inhibitors.

Conversion therapy is one of the therapeutic approaches indicated for unresectable HCC by the China National Health Commission's “Guidelines for the Diagnosis and Treatment of Hepatocellular Carcinoma (2019 Edition)” [15]. In our survey, 79% of surgeons and 19% of interventionalists would attempt downstaging/conversion therapy for patients with potentially resectable HCC (Figure 2a). “Failure to achieve R0 resection (63%),” “high tumor burden (26%),” and “portal vein tumor thrombus (21%)” were the main factors for experts to consider downstaging/conversion therapy. Moreover, 78% of experts preferred TKI + IO‐based therapies as the main downstaging/conversion therapeutic regimens for potentially resectable HCC, and 19% chose TKI combined with interventional therapy as the preferred treatment option (Figure 2b). Approximately 98% of experts indicated that the confirmed objective response rate (ORR) was an essential consideration in their selection of downstaging/conversion therapy, and safety was another important consideration. In our investigation, experts stated that 18% of the potentially resectable HCC patients were successfully downstaged/converted in their clinical practice. Approximately 67% of experts considered less than 1 month after successful downstaging/conversion therapy as the best time for surgery (Figure 2c).

In clinical practice, 62% of experts used adjuvant treatment after surgery after successful down‐staging therapy for all patients. Furthermore, 67% of the patients began adjuvant therapy within 1 month after the operation, and 29% began adjuvant therapy within 2 months after operation. Approximately 41% of experts indicated that the maintenance period of adjuvant therapy for patients with potentially resectable HCC was 6 months, while others indicated that the maintenance duration of patients with potentially resectable HCC was 12 months (Figure 2d). The maintenance duration for potentially resectable HCC patients was similar to that of resectable HCC patients.

### 3.4 TKI + IO‐Based Combined Therapies as the 1 L Treatment for Completely Unresectable HCC

**Figure 3:**
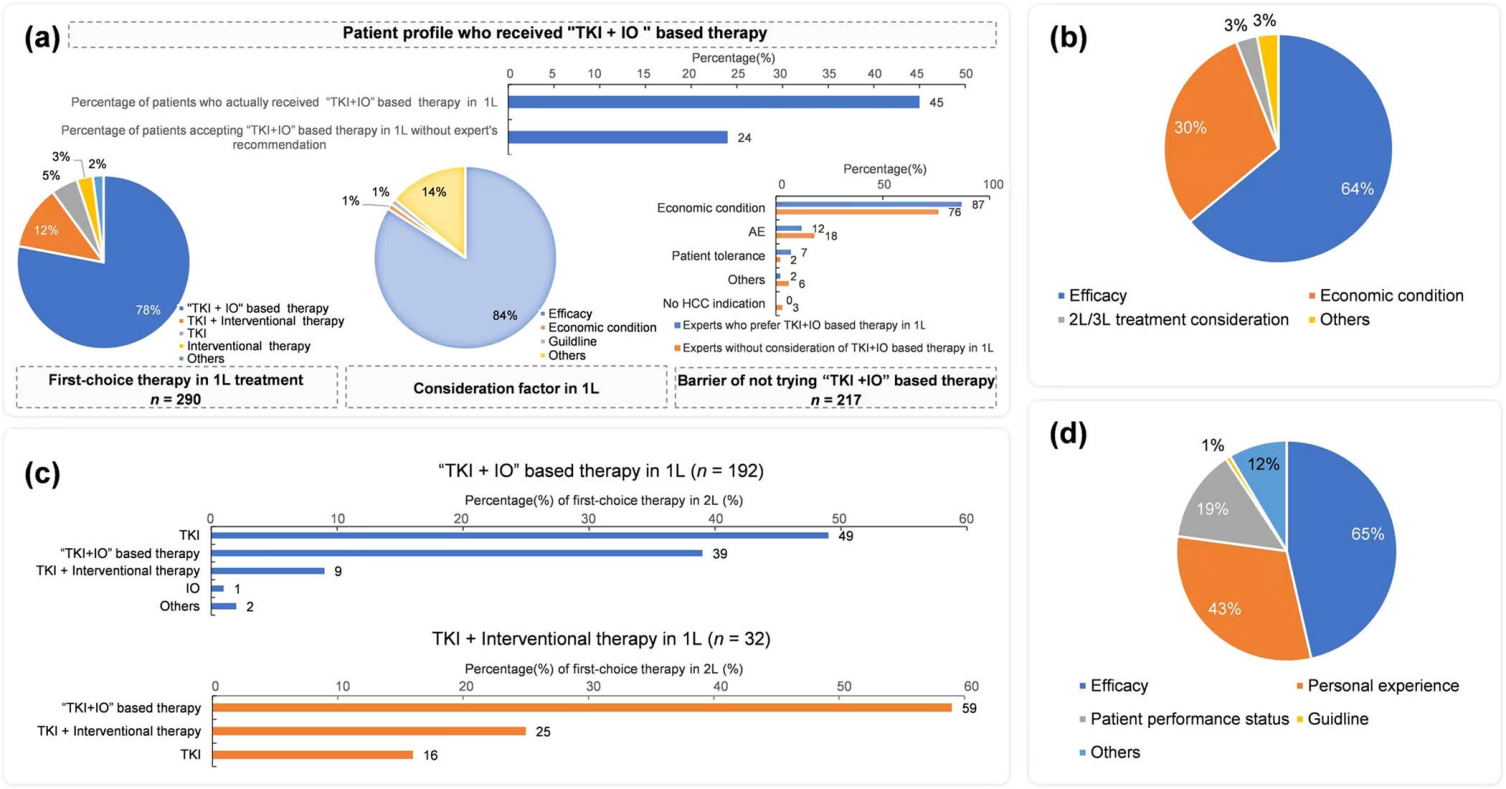
TKI + IO‐based combined therapies as the first‐line treatment for completely unresectable HCC. (a) The treatment concepts and clinical practice of TKI + Interventional therapy as the first‐choice in the first‐line treatment. (b) Consideration factor of choosing TKI + Interventional therapy in 1 L. (c) The treatment concepts of the first‐choice in the second‐line treatment for unresectable HCC patients. (d) The consideration of IO therapy in the second‐ and third‐line treatment for unresectable HCC patients. AE, adverse event; HCC, hepatocellular carcinoma; IO, immunotherapy; TKI, tyrosine kinase inhibitors; 1 L first‐line; 2L second‐line; 3 L, third line.

Approximately 78% of the experts stated that TKI + IO‐based combination therapy was the 1 L treatment for patients with completely unresectable HCC. Efficacy was the main reason for choosing this regimen. In clinical practice, only 45% of patients recommended for TKI + IO‐based combination therapy by experts selected TKI + IO‐based combination therapy as the 1 L therapy; the main reason for treatment rejection was “affordability” (Figure 3a). In clinical practice, among the patients whom experts recommended not to choose TKI + IO‐based combination therapy as the first choice, approximately 24% accepted TKI + IO‐based therapy as the 1 L therapy because of enrolling in a clinical study (Figure 3a). TKI + interventional therapy was recognized by 12% of experts as the 1 L therapy for completely unresectable HCC patients, and efficacy and affordability were the primary considerations (Figure 3b). For experts who considered TKI + IO‐based therapy as the preferred treatment in the 1 L therapy, 49% considered sequence TKI mono‐therapy and 39% considered a different MOA of TKI + IO regimen as the preferred 2 L treatment. Furthermore, experts considered TKI + interventional therapy as the preferred treatment for 1 L therapy. Most experts selected TKI + IO‐based treatment as the first choice for 2 L treatment (Figure 3c). Regardless of the 1 L therapy, 56% of experts might consider an IO‐based regimen (including IO monotherapy and IO combined with other treatments) as 2 L or higher therapy for completely unresectable HCC. The consideration for selecting this plan is shown in Figure 3d.

### 3.5 Changes in the Treatment Concepts and Clinical Practice for Resectable HCC

**Figure 4:**
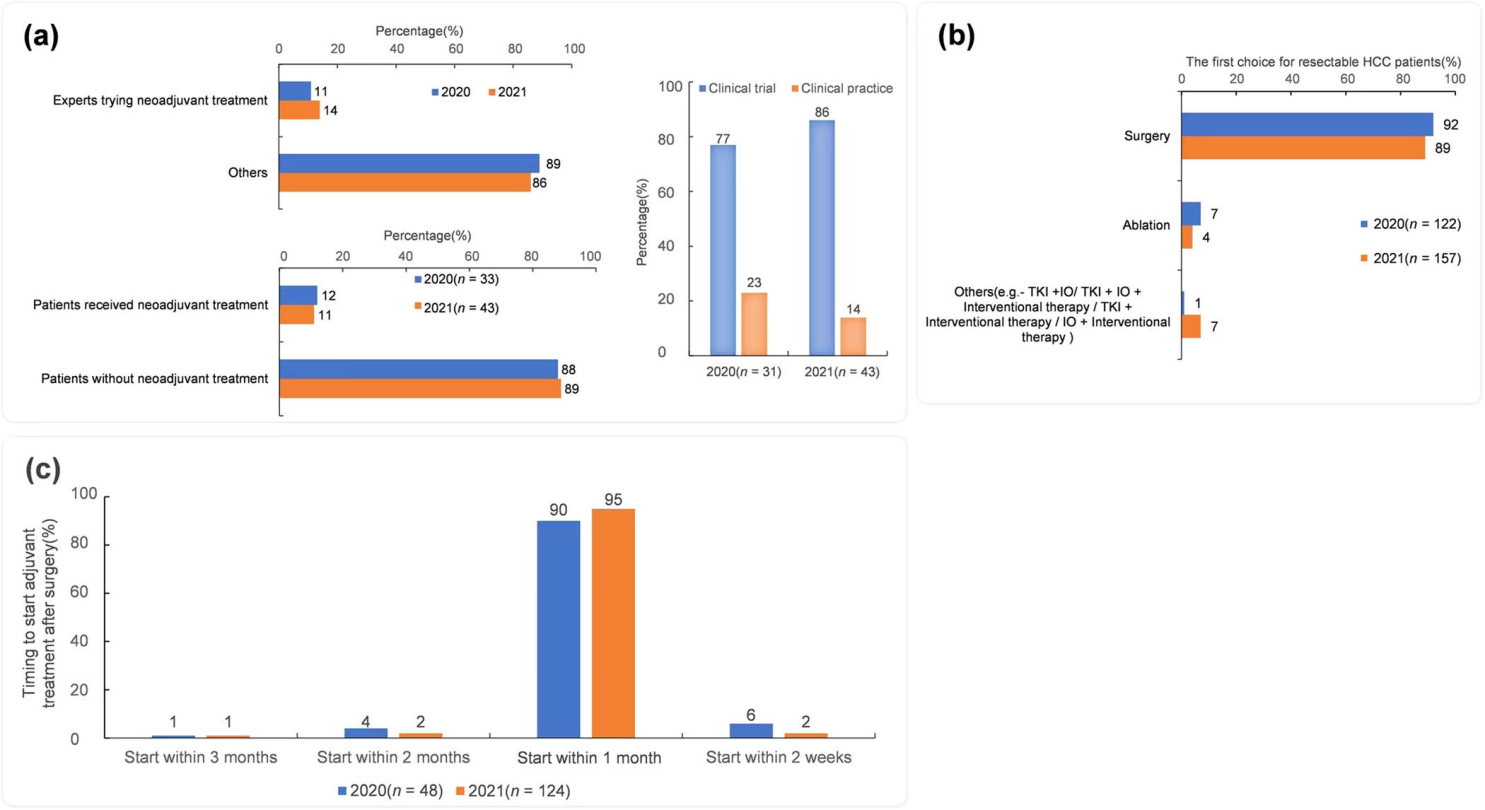
Changes in the tendency of treatment concepts and clinical practice for resectable HCC. (a) The treatment concepts and clinical practice of neoadjuvant treatment for resectable HCC patients. (b) The first choice for resectable HCC patients. (c) Timing to start adjuvant treatment after surgery. HCC, hepatocellular carcinoma; IO, immunotherapy; TKI, tyrosine kinase inhibitors.

More hepatobiliary surgeons recommended neoadjuvant therapy in 2021 compared with 2020 (11% to 14%), but the proportion of patients actually receiving the neoadjuvant therapy was similar. Data on neoadjuvant therapy still came from clinical trials (Figure 4a). For resectable HCC, surgery was still the primary treatment option, with no significant change between 2020 and 2021 (Figure 4b). In clinical practice, 41% of patients received adjuvant therapy to prolong disease‐free survival and overall survival (OS) in 2020, and this proportion remained unchanged in 2021. The reasons why these patients did not use adjuvant therapy included payment ability and adverse event management. The proportion of TKI + interventional therapy as the first choice for hepatobiliary surgeons increased from 31% in 2020 to 52% in 2021. More experts recommended that patients receive adjuvant therapy within 1 month after surgery in 2021 (Figure 4c). Notably, nearly 10% of experts selected systemic therapy ± locoregional therapy as the first choice for resectable HCC in 2021, indicating that with the development of systemic therapy, experts were more interested in exploring new methods.

### 3.6 Changes in the Treatment Concepts and Clinical Practice for Unresectable HCC

**Figure 5:**
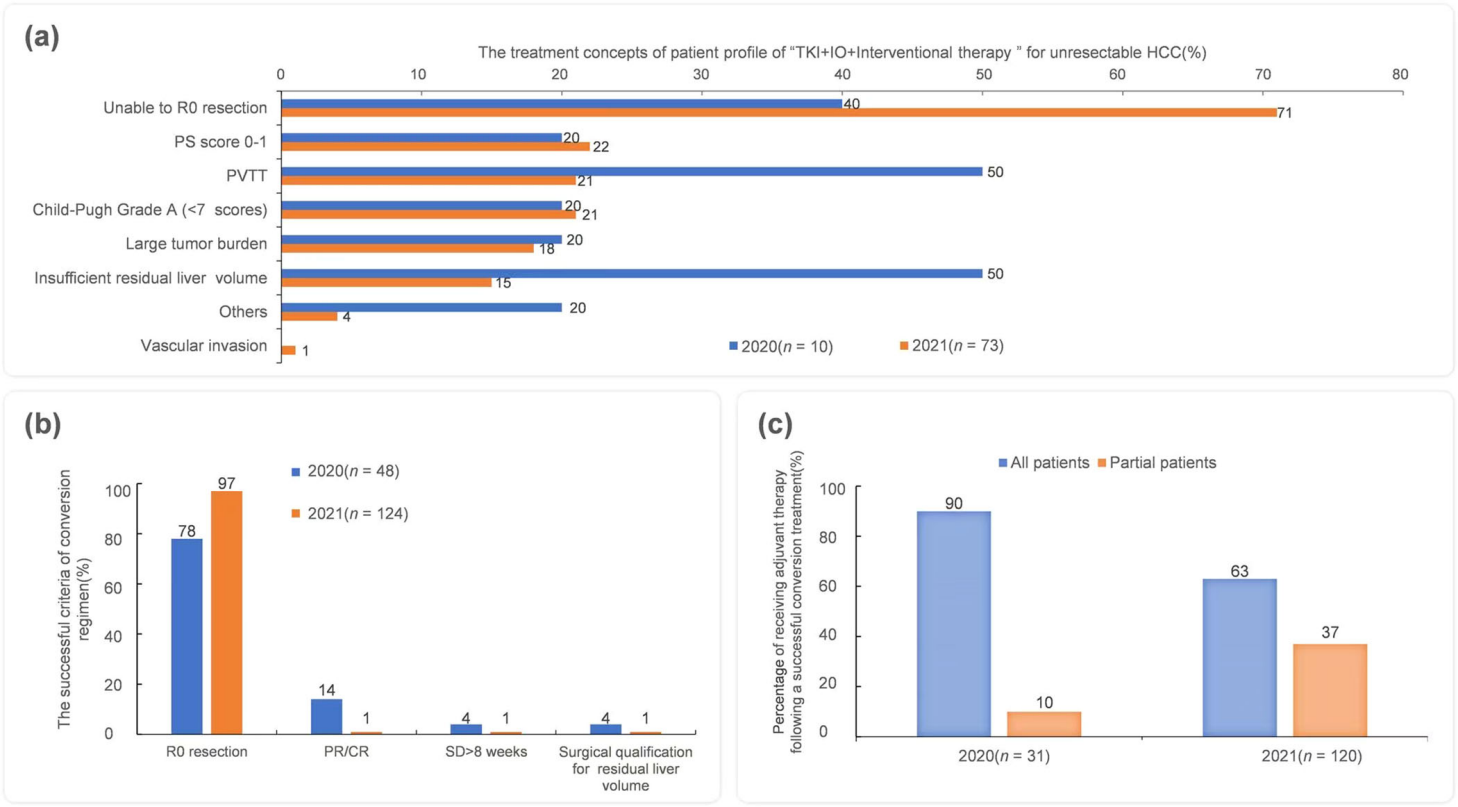
Changes in the tendency of treatment concepts and clinical practice for unresectable HCC. (a) The treatment concepts of patient profile of “TKI + IO + Interventional therapy” for unresectable HCC. (b) The successful criteria of conversion regimen and the percentage change of patients accepted adjuvant treatment subsequently to a successful conversion regimen. (c) Adjuvant treatment after surgery of successful conversion regimen. CR, complete response; PR, partial response; PVTT, portal vein tumor thrombosis; SD, stable disease.

Emerging evidence suggests that preoperative TKI + IO‐based therapy can give some unresectable HCC patients a chance of surgical resection, thus achieving long‐term survival [6]. More experts in 2021 (71% vs. 40%) recommended TKI + IO + interventional therapy as the first choice for “R0 unresectable” HCC patients. The experts also considered performance status, portal vein tumor thrombus, Child–Pugh score, tumor burden, and future liver residue (Figure 5a). In 2021, 97% of experts stated that “R0 resection” was a successful criterion for implementing a down‐staging/conversion regimen (Figure 5b). As the efficacy improved through TKI + IO‐based therapy, more patients achieved R0 resection, so more experts considered that only some patients needed adjuvant treatment. More experts in 2021 compared with 2020 considered “R0 resectable” as a down‐staging/conversion regimen successful criterion. In clinical practice, fewer patients in 2021 compared with 2020 (63% vs. 90%) accepted adjuvant treatment after surgery after a successful down‐staging/conversion regimen (Figure 5c).

### 3.7 Changes in the Treatment Concepts and Clinical Practice for Completely Unresectable HCC

**Figure 6:**
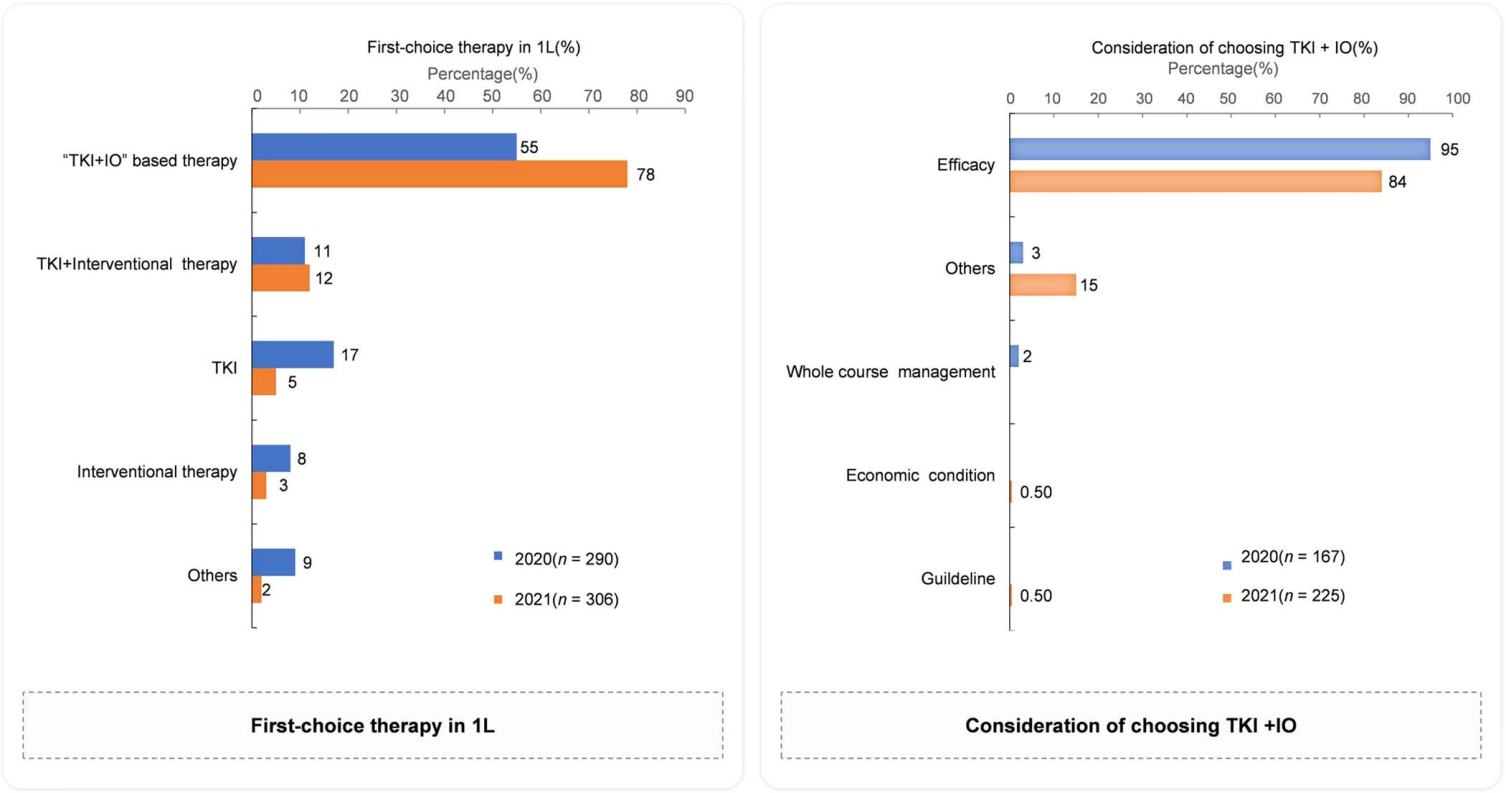
First choice therapy in the first‐line treatment for patients with unresectable HCC and consideration of choosing TKI + IO. IO, immunotherapy; TKI, tyrosine kinase inhibitors.

In this study, the proportion of experts indicating TKI + IO‐based therapy as their first choice for 1 L treatment increased from 55% in 2020 to 78% in 2021. The curative effect was still the primary consideration to choose TKI + IO‐based therapy as the first choice for the 1 L treatment. Furthermore, in 2021, approximately 15% of experts considered factors including converting to resectable status, clinical study enrollment, and other factors (Figure 6). In 2020, 49% of patients tried TKI + IO‐based treatment as the first choice for 1 L treatment, and the proportion was similar (at 45%) in 2021.

## 4 Discussion

In recent years, rapid progress in systemic therapies has led to the approval of many therapeutic methods that have quickly changed clinical guidelines and practices. Because of the high heterogeneity of HCC, there are still some gaps between real‐world clinical practice and guidelines [16]. Therefore, a comprehensive understanding of the current treatment concepts and clinical practice for HCC in China is needed. We conducted a questionnaire survey on hundreds of HCC experts in China to analyze and evaluate the treatment concepts and clinical practice for HCC in China in 2020 and 2021.

Consistent with previous reports [17], the survey results indicated that 39% of HCC cases were resectable when first diagnosed, and surgical resection was the primary treatment choice of experts for resectable HCC patients in 2021. In recent years, comprehensive neoadjuvant and adjuvant therapy for HCC, such as immunotherapy and TKI drugs, has achieved remarkable curative effects and improved the survival and quality of life of patients with resectable HCC. High‐level evidence to guide treatment decision‐making is lacking. The feasibility of neoadjuvant therapy has been proven in other solid organ malignant tumors. However, a consensus has not been reached regarding the effects of neoadjuvant therapy on HCC [18]. In our survey, approximately 28% of experts would indicate neoadjuvant treatment for resectable HCC patients before surgery. However, in clinical practice, only 11% of patients received neoadjuvant treatment. Notably, the proportion of experts who would indicate postoperative adjuvant treatment for resectable HCC patients was approximately 69% and the proportion of patients who received postoperative adjuvant treatment was approximately 41%. These differences may be from poor patient compliance, no clear recommendations from guidelines, and poor patient tolerance. However, the experts indicated that patients with high‐risk recurrence factors, such as MVI, high AFP, multiple nodules, and incomplete tumor capsule, were the main targets of neoadjuvant/adjuvant therapy [19]. Disease progress may develop in some patients during neoadjuvant therapy, resulting in missed surgical opportunities. Therefore, it is necessary to screen people who are suitable for neoadjuvant therapy [20]. Moreover, long‐term drug use will not only increase the economic burden but also lead to cumulative toxicity [21]. The survey results indicated that treatment concepts in different regions and institutions were diverse. In general, teams with extensive experience in combined therapy and good management of adverse events widely administer combined therapy.

Downstaging/conversion therapy plays an important role in the treatment of unresectable liver cancer as a part of nonoperative treatment of liver cancer [21, 22, 23, 24]. Reports on the down‐staging/conversion therapy for HCC are increasing, with promising results [25]. Our survey focused on the changes brought about by advances in systematic therapy to downstaging/conversion therapy. For potentially resectable HCC, 79% of hepatobiliary surgeons and 19% of interventional physicians were willing to choose down‐staging/conversion therapy. TKI + IO‐based treatment (78%) was the main downstaging/conversion scheme, and ORR (98%) was the most important consideration. Approximately 19% of experts selected TKI + IO, considering both ORR and safety. Moreover, 97% thought “R0 resection” was the main criterion for a successful down‐staging/conversion regimen. Our results showed that the mean successful down‐staged rate was approximately 18% in clinical practice. The success rates of different down‐staging/conversion treatments vary greatly, which may be related to different participants or other factors [22, 23, 24, 25]. Down‐staging/conversion therapy of HCC has been gradually recognized as an important treatment for advanced and unresectable HCC. However, data on this treatment are limited, and more high‐level clinical evidence is needed to form a standardized implementation standard. At present, whether to use adjuvant therapy after conversion is controversial and mainly requires guidance of tumor pathological examination. According to the discussion and opinions of domestic experts, the preliminary recommendations of the postoperative plan are made, IO alone or TKI + IO should be continued for 6–12 months after surgery based on specific postoperative pathology results [26]. Currently, many studies on downstaged/conversion therapy have been carried out in China and worldwide, but most are retrospective studies at a single center with a small sample size [27, 28]. Prospective multicenter clinical studies with large sample sizes are needed to provide evidence to generate guidelines to better guide clinical practice.

While progress in medical treatment has improved the prognosis of HCC, the survival of HCC patients after recurrence is still not ideal. The risk factors of early HCC recurrence are mainly related to tumor size, tumor number, microvascular infiltration, and other tumor load and biological characteristics; late recurrence is mainly related to the degree of liver cirrhosis, HBV DNA, and other liver inflammatory background factors [29]. At present, postoperative adjuvant transcatheter arterial chemoembolization (TACE) is the most common treatment for patients with high‐risk recurrence. The results of a randomized controlled study showed that for patients with a tumor length > 5 cm, microvascular invasion, and multiple liver cancer, adjuvant TACE therapy for approximately 1 month after operation significantly prolonged tumor‐free survival and OS [30]. The guidelines of the Chinese Society of Clinical Oncology recommend routine TACE treatment for patients with residual lesions, multiple nodules, tumor diameter > 5 cm, and vascular invasion [31]. In this study, 64% of experts would choose TACE as postoperative adjuvant treatment, followed by TKI + TACE and TKI. Research data were the main consideration for experts to choose interventional treatment and TKI. Personal experience was also an important consideration factor when experts chose IO therapy. The duration of postoperative adjuvant treatment varies from center to center [32]. Our findings indicated that 95% of experts chose to start adjuvant treatment within 1 month after the operation. Most experts stated that postoperative adjuvant treatment should last for 6 or 12 months, but there is no evidence‐based guideline or consensus. The STORM study showed that there was no significant difference in median recurrence‐free survival between the sorafenib group and the placebo group, but in‐depth analysis of the data found that nearly 50% of the patients had single tumors without microvascular invasion, and the risk of metastasis and recurrence was relatively low [33]; this result does not deny the potential value of targeted drugs in postoperative adjuvant therapy. Additionally, postoperative targeted therapy for patients with high‐risk recurrence should be based on postoperative TACE therapy. Whether targeted therapy combined with TACE can reduce the postoperative recurrence rate of liver cancer is also worth further investigation. Keynote 937 is a Phase III study that will evaluate the safety and efficacy of pembrolizumab versus placebo as adjuvant therapy in participants with HCC and complete radiological response after surgical resection or local ablation. Such research will provide new answers for adjuvant therapy.

Regarding the preferred treatment scheme for neoadjuvant treatment, approximately 10% of the experts in 2021 would select systematic treatment ± locoregional therapy; the interest in exploring neoadjuvant therapy was increasing with the development of systematic treatment. In this survey, the proportion of experts who selected TKI + IO ± locoregional therapy as the 1 L therapy increased from 55% in 2020 to 78% in 2021. After the development of TKI + IO, the proportion of experts who switched to recommending a single TKI increased. For 2 L and later treatments, the proportion of doctors who considered IO monotherapy or IO combination therapy increased from 9% in 2020 to 28% in 2021. For unresectable HCC, as for the criteria for a successful conversion, fewer experts mentioned the need for partial response/complete response in 2021. The proportion of experts who stated that all patients undergoing surgical resection after down‐staging/conversion treatments needed postoperative adjuvant therapy decreased from 90% to 63%.

The reasons for the changes in treatment concepts and clinical practice include the following: (1) clinical research results are diverse, with success and failure of studies, and influence the doctor's approach and practice; and (2) changes in the concepts and practice of experts will also influence the design of future clinical studies 1 L.

Marked changes in the approaches of Chinese doctors towards HCC are being seen along with expansions in treatment methods, which has led to advanced liver cancer patients achieving an OS of over 20 months. These improvements have led to new challenges in subsequent scientific research, and understanding the impact of 2 L therapy on the OS of patients and the results of experimental studies is critical. Moreover, doctors are facing more challenges regarding the choice of treatment methods, including how to perform patient stratification, which patients benefit from a single targeted treatment, and which should undergo combined immunotherapy. Moreover, how to select the appropriate treatment sequence, the 1 L treatment plan, and 2 L treatment plan are critical questions. Evidence‐based studies are needed to answer these questions.

This study has several limitations. First, surgical abilities were diverse at different hospitals, and the disease severity of patients may vary across different regions. These factors, along with the various educational backgrounds of patients and their ability to afford treatment, might influence the treatment concepts and clinical practice of doctors and lead to bias. During the survey, there may be instances where oncologists respond to questions relating to the surgical field (e.g., oncologists answering questions related to perioperative issues). Additionally, because the analysis was descriptive in nature, the potential influence of these factors has not been eliminated through statistical modeling, which may lead to bias. However, the selected experts included associate professors and above from tertiary hospitals in most provincial capitals across China, providing a certain level of representativeness. Furthermore, we implemented quality control measures, such as excluding expert data with response rates below 80%. Analysis of the survey results was delayed to a certain extent because of the COVID‐19 pandemic, but the results still reflect the changing trend of the concepts of treatment. Another well‐designed large‐scale survey is ongoing, and the results will be published next year.

## 5 Conclusion

Surgical resection is a necessary treatment for resectable HCC. For unresectable or advanced HCC, 1 L or 2 L therapy based on TKI + IO provides favorable antitumor activity and good clinical efficacy. The comprehensive multidisciplinary treatment of HCC brings hope to patients.

## Author Contributions

**Hong Zhao:** conceptualization (equal), data curation (equal), formal analysis (equal), investigation (equal), methodology (equal), writing – original draft (equal), writing – review and editing (equal). **Yilei Mao:** conceptualization (supporting), software (supporting), supervision (lead), validation (supporting), writing – original draft (supporting), writing – review and editing (supporting). **Hongguang Wang:** data curation (supporting), investigation (lead), software (supporting), supervision (supporting), visualization (supporting), writing – review and editing (supporting). **Aiping Zhou:** formal analysis (supporting), investigation (supporting), methodology (supporting), project administration (supporting), writing – original draft (equal), writing – review and editing (supporting). **Zhengqiang Yang:** conceptualization (supporting), data curation (supporting), formal analysis (supporting), software (supporting), supervision (supporting), validation (supporting), visualization (supporting). **Yue Han:** project administration (supporting), resources (supporting), software (supporting), supervision (supporting), validation (supporting), writing – review and editing (supporting). **Gong Li:** methodology (supporting), project administration (supporting), validation (supporting), visualization (supporting), writing – original draft (supporting), writing – review and editing (supporting). **Xinyu Bi:** data curation (supporting), formal analysis (lead), investigation (supporting), resources (supporting), software (lead). **Chunyi Hao:** resources (supporting), software (supporting), validation (supporting), visualization (supporting), writing – original draft (lead), writing – review and editing (lead). **Xiaodong Wang:** investigation (supporting), validation (supporting), visualization (lead), writing – original draft (supporting), writing – review and editing (supporting). **Jun Zhou:** data curation (supporting), formal analysis (supporting), resources (supporting), software (supporting), supervision (lead). **Chaoliu Dai:** data curation (supporting), formal analysis (supporting), resources (supporting), software (supporting), supervision (supporting). **Feng Wen:** methodology (supporting), project administration (supporting), visualization (supporting), writing – original draft (supporting), writing – review and editing (supporting). **Jingdong Zhang:** data curation (supporting), resources (supporting), software (supporting), supervision (supporting), validation (supporting). **Ruibao Liu:** conceptualization (supporting), data curation (supporting), formal analysis (supporting), resources (supporting), software (supporting). **Tao Li:** methodology (supporting), project administration (supporting), visualization (supporting), writing – original draft (supporting), writing – review and editing (supporting). **Lei Zhao:** conceptualization (supporting), data curation (supporting), resources (supporting), software (supporting), supervision (supporting). **Zuoxing Niu:** conceptualization (supporting), data curation (supporting), resources (supporting), software (supporting), supervision (supporting). **Tianfu Wen:** conceptualization (supporting), data curation (supporting), resources (supporting), software (supporting), supervision (supporting). **Qiu Li:** conceptualization (supporting), data curation (supporting), resources (supporting), software (supporting), supervision (supporting). **Hongmei Zhang:** conceptualization (supporting), data curation (supporting), resources (supporting), software (supporting), supervision (supporting). **Xiaoming Chen:** conceptualization (supporting), data curation (supporting), resources (supporting), software (supporting), supervision (supporting). **Minshan Chen:** conceptualization (supporting), data curation (supporting), resources (supporting), software (supporting), supervision (supporting). **Ming Zhao:** conceptualization (supporting), data curation (supporting), formal analysis (supporting), validation (supporting), visualization (supporting). **Yajin Chen:** conceptualization (supporting), data curation (supporting), resources (supporting), software (supporting), supervision (supporting). **Jun Yu:** resources (supporting), software (supporting), supervision (supporting), validation (supporting), visualization (supporting). **Jie Shen:** resources (supporting), software (supporting), supervision (supporting), validation (supporting), visualization (supporting). **Xiangchen Li:** resources (supporting), software (supporting), supervision (supporting), validation (supporting), visualization (supporting). **Lianxin Liu:** resources (supporting), software (supporting), supervision (supporting), validation (supporting), visualization (supporting). **Zhiyong Huang:** resources (supporting), software (supporting), supervision (supporting), validation (supporting), visualization (supporting). **Wei Zhang:** resources (supporting), software (supporting), supervision (supporting), validation (supporting), visualization (supporting). **Feng Shen:** resources (supporting), software (supporting), supervision (supporting), validation (supporting). **Weiping Zhou:** resources (supporting), software (supporting), supervision (supporting), validation (supporting). **Zhengang Yuan:** conceptualization (supporting), data curation (supporting), resources (supporting), software (supporting), supervision (supporting). **Jian Zhai:** resources (supporting), software (supporting), supervision (supporting), validation (supporting). **Ningling Ge:** data curation (supporting), formal analysis (supporting), resources (supporting), software (supporting), supervision (supporting). **Yongjun Chen:** resources (supporting), software (supporting), supervision (supporting), validation (supporting), visualization (supporting). **Huichuan Sun:** resources (supporting), software (supporting), supervision (supporting), validation (supporting), visualization (supporting). **Jianqiang Cai:** conceptualization (equal), data curation (equal), formal analysis (equal), resources (equal), software (equal), supervision (equal), validation (equal).

## Ethics Statement

The authors have nothing to report.

## Consent

Informed consent was obtained from all experts surveyed in this study.

## Conflicts of Interest

The authors declare no conflicts of interest.

## Data Availability

All data generated or analyzed during this study are included in this published article.
